# Causal Relationship Between Glycated Hemoglobin (HbA1c) and Cataract: A Bidirectional Mendelian Randomization Study

**DOI:** 10.1167/tvst.14.9.23

**Published:** 2025-09-16

**Authors:** Guoqing Wu, Xin Kao

**Affiliations:** 1 Department of Ophthalmology, Zhengda Guangming Eye Group, Weifang Weicheng Zhengda Guangming Eye Hospital, Weifang City, Shandong Province, China; 2 Department of Ophthalmology, Zhengda Guangming Eye Group, Weifang Eye Hospital, Weifang City, Shandong Province, China

**Keywords:** bidirectional mendelian randomization, HbA1c, cataract, enrichment analysis, validation analysis

## Abstract

**Purpose:**

Glycated hemoglobin (HbA1c) has been linked to cataract in previous epidemiological observational studies; however, the exact causal relationship between HbA1c and cataract formation remains unclear.

**Methods:**

We performed a bidirectional Mendelian randomization (MR) analysis using data from two datasets in IEU OpenGWAS database. Inverse-variance weighted (IVW) estimation was used as the primary analysis, followed by the application of four complementary methods to detect and correct the effect of horizontal pleiotropy. Single nucleotide polymorphisms (SNPs) were used as instrumental variables (IVs) for HbA1c or cataract. Comparable results were obtained by methods such as Cochran's Q. Enrichment analysis was performed on IV-associated genes. Validation analysis based on cell lines was performed to reveal the effect of HbA1c in vitro.

**Results:**

A total of 183 SNPs were used as significant IVs for HbA1c in forward MR analysis. The results showed that HbA1c was significantly associated with cataracts (odds ratio = 1.022, 95% confidence interval, 1.012-1.031, *P* = 2.95E-06). However, the causal effect of cataracts on HbA1c was not significant in the reverse MR analysis. The IVW method provided the ideal results in current bidirectional MR analysis. Horizontal pleiotropy was unlikely to distort the causal estimates according to the sensitivity analysis. IV-associated genes were mainly enriched in carbohydrate metabolism function and autophagy pathway. Elevated HbA1c levels significantly increased ROS production, reduced SOD activity, and induced apoptosis.

**Conclusions:**

Findings of this MR study supported a causal effect of HbA1c on cataract, underscoring the clinical importance of tight glycemic control as a strategy for reducing cataract risk.

**Translational Relevance:**

With the further study of HbA1c, this study suggests that there is a causal relationship between HbA1c and cataract and suggests that strict blood glucose control can be a strategy to reduce the risk of cataracts.

## Introduction

Cataracts, marked by the clouding of the eye's lens, is the foremost cause of blindness globally.^1^ Although age remains the most significant risk factor, other contributors such as diabetes, ultraviolet radiation exposure, and smoking have been implicated in cataractogenesis.^2^^,^^3^ Among these risk factors, diabetes, particularly type 2 diabetes, has garnered considerable attention because of its growing prevalence and established association with cataracts.^4^ Notably, glycemic control, often assessed through glycated hemoglobin (HbA1c) levels, is a critical component of diabetes management and has been linked to various diabetic complications.^5^^,^^6^ However, the exact causal relationship between HbA1c and cataract formation remains unclear.

HbA1c is a widely recognized biomarker reflecting average blood glucose levels over a period of approximately three months.^7^^,^^8^ Elevated HbA1c is not only indicative of poor glycemic control but also serves as a predictor of microvascular complications, including retinopathy and nephropathy.^9^ Despite extensive epidemiological evidence suggesting a potential link between higher HbA1c levels and cataract development, establishing a clear causal relationship remains challenging because of potential confounding factors such as age, lifestyle, and coexisting medical conditions.^10^ Numerous observational studies have found a link between diabetes-related hyperglycemia and the accelerated development of cataracts. However, these findings may be confounded by reverse causality, in which individuals with pre-existing cataracts or other comorbidities may experience worsened glycemic control because of reduced physical activity or limited access to healthcare.^11^ Moreover, a lack of randomized controlled trials (RCTs) addressing this specific relationship leaves a significant gap in our understanding of whether HbA1c directly influences cataract formation, or whether the association is primarily due to shared risk factors. Mendelian randomization (MR) is an instrumental variable approach that uses single-nucleotide polymorphisms (SNPs) as instrumental variables (IVs) to deduce causal relationships between two traits.^12^ MR mainly uses the influence of genetically determined variation on phenotype to infer the influence of phenotype on disease. The principle is grounded in Mendelian inheritance principles, and alleles follow the principle of random distribution during gamete formation.^13^ MR allows genetic variants of known influencing factors, such as alcohol consumption or low-density lipoprotein levels, to be used as instrumental variables, allowing insight into the effects of alcohol consumption on pregnancy and low-density lipoprotein levels on cardiovascular disease.^14^ MR is methodologically analogous to randomization in RCTs, with their results often aligning and providing evidence for drug target verification.^15^ Recent MR studies have demonstrated causal effects of HbA1c on diverse health outcomes, including eye disease^16^ and diabetic complications,^17^ thereby establishing a methodological foundation for investigating cataractogenesis. For example, Jiang et al. employed two-sample MR analysis to assess causal effects of clinical or behavioral factors on cataract risk. Their findings revealed significant associations between genetically determined primary open-angle glaucoma and mean spherical equivalent myopic refractive error with cataract risk, suggesting that primary open-angle glaucoma and myopia may be contribute to age-related cataractogenesis.^18^ Georgakis et al.^19^ conducted MR analysis and found that genetic susceptibility to elevated T2D and HbA1c levels was associated with the high risk of ischemic stroke, large-artery stroke, and small vessel stroke, supporting a causal effect of T2D and hyperglycemia on these stroke subtypes.

In this study, we used a bidirectional MR design to examine the possible causal link between HbA1c levels and the risk of cataracts. By applying various estimation methods, we sought to assess both the direction and strength of the causal relationship between these two variables. Our findings have the potential to inform future clinical strategies for cataract prevention, particularly in patients with diabetes, and to deepen our understanding of the biological mechanisms underlying the development of cataracts under hyperglycemic conditions.

## Methods

### Data Sources

The data sources for HbA1c (GWAS ID: ukb-d-30750_raw) and cataract (GWAS ID: ebi-a-GCST90018814) were taken from the IEU OpenGWAS database. There were 344182 samples (population: European) and 491877 samples (population: European) in HbA1c data and cataract data, respectively. The HbA1c data included 344182 samples (population: European) and 13,586,180 SNPs, whereas the cataract data included 491,877 samples (population: European) and 24,163,031 SNPs.

### The Optimal IV Selection

In the current MR analyses, suitable instrumental variables (SNPs) for HbA1c and cataract were identified using the TwoSampleMR (version: 4.3.2). Specifically, genetic variants (SNP) were chosen according to a threshold for significance across the genome (*P* < 5 × 10⁻⁸). Subsequently, appropriate SNPs were retained while accounting for linkage disequilibrium, defined by an *r*^2^ threshold greater than 0.001 (clumping distance = 10,000 kb). During the standardization of genetic data between exposure and outcome groups, SNPs exhibiting strand alignment discrepancies and intermediate allele frequencies were systematically excluded. Finally, *F*-statistics were calculated to assess the strength of the instruments, with SNPs demonstrating an *F*-statistic > 10 classified as strong IVs.

### MR Analyses

The IVW method was primarily used for the key causal estimates in our MR analyses, whereas four additional methods, including simple mode, weighted mode, weighted median, and MR-Egger regression, were used to enhance the IVW estimates because they offer more robust results across various scenarios.^20^ *P* < 0.05 from the IVW analysis, coupled with consistent directional results from the other four methods, indicated statistical significance for causal inference. The findings were illustrated through scatter plots and forest plots. Additionally, the Cochran's Q test was conducted to evaluate heterogeneity based on the MR-Egger and IVW methods. For significant estimates, we further investigated horizontal pleiotropy using MR-Egger regression and MR_PRESSO analysis, with a cutoff of *P* > 0.05 suggesting the absence of horizontal pleiotropy. Finally, a leave-one-out test was performed for sensitivity analysis.^21^

### Enrichment Analysis for IV-Associated Genes

In the main analyses (forward MR), we used cis-eQTL data to investigate genes associated with the instrumental variables. Subsequently, GO function and KEGG pathway analyses were conducted on these genes using a significance level of *P* < 0.05. This investigation would help to reveal whether SNPs (IVs) were associated with changes in the expression of specific genes and further speculation whether these genes played roles in the relationship between HbA1c and cataracts.

### Validation Analysis

The cell experiment was performed in the current study to validate the effect of HbA1c on cataracts. Briefly, the human lens epithelial cells (HLECs, ATCC, CRL-11421) were cultured in Dulbecco's modified Eagle medium (DMEM) with high glucose (4.5 g/L glucose; 11965092; Gibco, Thermo Fisher Scientific, Waltham, MA, USA), supplemented with 10% fetal bovine serum (10099141; Gibco) and 1% penicillin-streptomycin (15140122; Gibco). To simulate a hyperglycemic environment, we designed a total of three groups: the control group (HLECs were cultured in normal DMEM with a glucose concentration of 5.5 mM), the high-glucose group (HLECs were cultured in DMEM with a glucose concentration of 30 mM), and the HbA1c group (HLECs were cultured in high-glucose DMEM [30 mM] with the addition of 50 µg/mL glycated hemoglobin [HbA1c, H9892; Sigma-Aldrich Corp., St. Louis, MO, USA] to mimic prolonged elevated levels of glycated hemoglobin).

Cells were incubated for 72 hours in a CO_2_ incubator (Thermo Fisher Scientific, Heracell VIOS 160i) at 37°C with 5% CO_2_. Throughout this incubation, we performed a series of assays at various time points, including assessments of cell viability, oxidative stress, antioxidant enzyme activity, and apoptosis. The cell viability for each group was assessed with the CCK-8 assay (CK04-11; Dojindo Molecular Technologies, Inc., Kumamoto, Japan), with absorbance measured on a Synergy H1 microplate reader (BioTek Instruments, Agilent, Santa Clara, CA, USA) to analyze the effects of HbA1c on cell proliferation and survival. Furthermore, intracellular levels of ROS were quantified using a 2′,7′-dichlorofluorescin diacetate (DCF-DA) fluorescent probe (D6883; Sigma-Aldrich Corp.). Fluorescence intensity, corresponding to ROS levels, was measured by the Synergy H1 microplate reader. The activity of antioxidant enzymes was measured to evaluate the oxidative stress response. SOD activity was measured using ‌a commercial SOD Activity Assay Kit (S0101M; Beyotime Institute of Biotechnology, Jiangsu, China). Apoptotic cells were measured using the Annexin V-FITC/PI Apoptosis Detection Kit (556547; BD Bioscience, Franklin Lakes, NJ, USA) and analyzed by flow cytometry on a FACSCanto II flow cytometer (BD Bioscience), to investigate whether HbA1c increased apoptosis in HLECs.

Each experiment was performed three times, with outcomes presented as the mean ± standard deviation. Differences between groups were analyzed using one-way analysis of variance, considering *P* < 0.05 as the threshold for statistical significance.

## Results

### The Causal Effects of HbA1c on Cataracts

**Figure 1.**
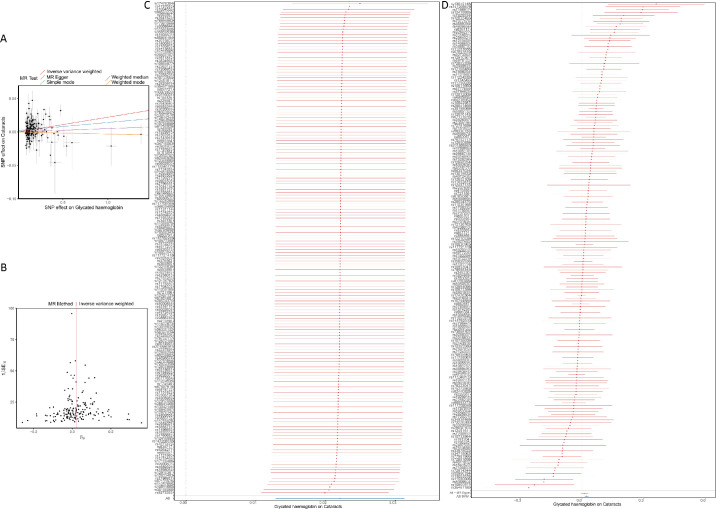
The forward MR analyses: casual effect of glycated hemoglobin (HbA1c) on cataract. (**A**) Scatter plot of the causal relationships between HbA1c and cataract using different MR methods: the slope of each line corresponding to the causal estimates for each method; individual SNP-effect on the outcome (*point and vertical line*) against its effect on the exposure (*point and horizontal line*) was delineated in the background. (**B**) Funnel plot analyses to evaluate sensitivity. (**C**) Leave-one-out analyses to evaluate sensitivity: all SNP results were on the right side of 0, indicating that our analysis results were not sensitive. (**D**) Forest plot was used to show the MR estimate and 95% CI values (*gray line segment*) for each SNP, also showed the IVW and MR-Egger MR results at the bottom.

A total of 183 independent SNPs with significant *P* < 1 × 10^−^^8^ were used as IVs for HbA1c in the forward MR analyses (Table 1). As shown in Figure 1A, the IVW result suggested that genetically predicted HbA1c was significantly associated with cataracts (odds ratio [OR] = 1.022; 95% confidence interval [CI], 1.012–1.031; *P* = 2.95E-06). The funnel plot showed a relatively uniform dispersion of points, which further indicated that our analysis results were not sensitive (Fig. 1B). Subsequently, the leave-one-out analysis was used to conduct sensitivity analysis on the IVs. The results indicated that the association remained stable in leave-one-out analyses, as removing any individual SNP did not significantly alter the observed relationship (Fig. 1C). Additionally, the combined *P* value from Cochran's Q test demonstrated significant heterogeneity (Fig. 1D). Despite the presence of heterogeneity, the IVW analysis yielded *P* < 0.05, indicating that the heterogeneity in the HbA1c and cataracts datasets did not significantly impact the results. Therefore, the findings of the analysis remained robust and valid. Furthermore, MR-Egger regression (global test *P* = 0.208) and MR-PRESSO (*P* = 0.292) revealed no indication of horizontal pleiotropy among SNPs used in the causal estimates (Table 2).

**Table 1.** The Forward MR Analysis for HbA1c on Cataracts

Outcome	Exposure	Method	NSNP	Beta	SE	P-Value	OR
Cataracts	HbA1c	MR-Egger	172	0.012053	0.009195	0.191662	1.012126
Cataracts	HbA1c	Weighted median	172	0.00499	0.006421	0.437063	1.005003
Cataracts	HbA1c	Inverse variance weighted	172	0.022032	0.004713	2.95E-06	1.022277
Cataracts	HbA1c	Simple mode	172	−0.00315	0.014118	0.823704	0.996855
Cataracts	HbA1c	Weighted mode	172	−0.00315	0.007381	0.670059	0.996855

*P* < 0.05 was considered as significant causal relationship.

**Table 2.** The Results of Sensitivity Analysis in Forward MR Analysis

			Heterogeneity Test			Pleiotropy Test			
Outcome	Exposure	Method	Q	Q_df	Q_pval	Egger Intercept	SE	pval	MR-PRESSO_pval
Cataracts	HbA1c	MR-Egger	331.2267	170	1.82E-12	0.002088	0.001653	0.208227	0.291662
Cataracts	HbA1c	Inverse variance weighted	334.336	171	1.18E-12	NA		NA	

pval, *P* value.

### The Causal Effects of Cataracts on HbA1c

**Figure 2.**
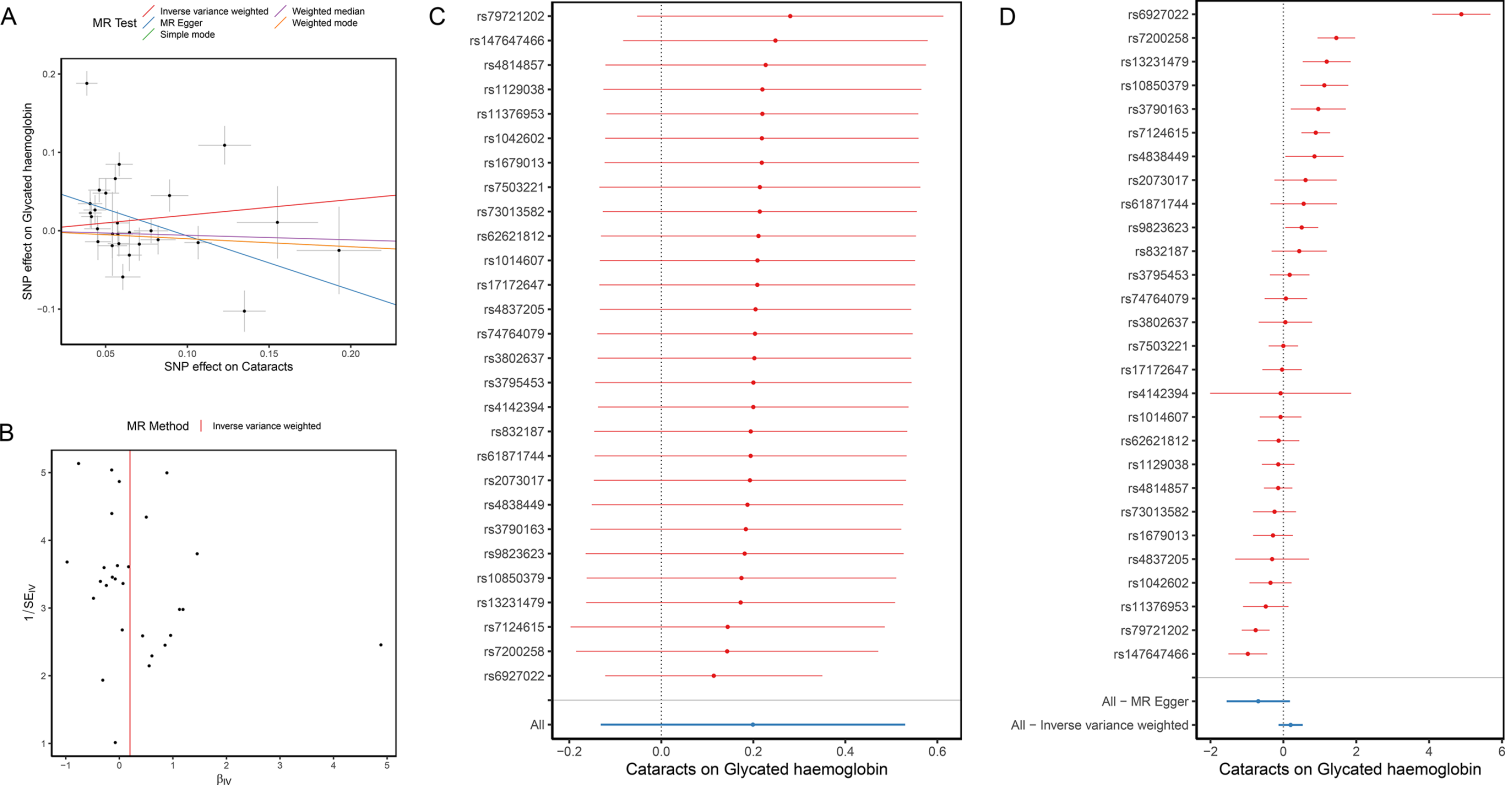
The reverse MR analyses: casual effect of cataract on HbA1c. (**A**) Scatter plot of the causal relationships between cataract and HbA1c using different MR methods: the slope of each line corresponding to the causal estimates for each method; individual SNP-effect on the outcome (*point and vertical line*) against its effect on the exposure (*point and horizontal line*) was delineated in the background. (**B**) Funnel plot analyses to evaluate sensitivity. (**C**) Leave-one-out analyses to evaluate sensitivity: all SNP results were on the right side of 0, indicating that our analysis results were not sensitive. (**D**) Forest plot was used to show the MR estimate and 95% CI values (*gray line segment*) for each SNP, also showed the IVW and MR-Egger MR results at the bottom.

In the reverse MR analyses, a total of 28 independent SNPs with significant *P* < 1 × 10^−^^8^ were selected as IVs for cataracts (Table 3). As illustrated in Figure 2A, the IVW analysis indicated that genetically predicted HbA1c was not significantly associated with cataracts (OR = 1.220; 95% CI, 0.876−1.698; *P* = 0.237). The funnel plot showed a relatively uniform dispersion of points, which further indicated that the analysis results were not sensitive (Fig. 2B). Subsequently, the leave-one-out analysis was used to conduct sensitivity analysis on the IVs. The findings indicated that the association remained consistent in leave-one-out analysis, because removing any individual SNP did not lead to significant changes (Fig. 2C), demonstrating the robustness of the results. Cochran's Q test indicated that the observed association exhibited significant heterogeneity (Fig. 2D). Additionally, significant horizontal pleiotropy among SNPs in the causal estimates was demonstrated by the MR-Egger regression (global test *P* < 0.05) and MR-PRESSO analysis (*P* < 0.001) (Table 4).

**Table 3.** The Reverse MR Analysis for Cataracts on HbA1c

Outcome	Exposure	Method	NSNP	Beta	SE	*P* Value	OR
HbA1c	Cataracts	MR-Egger	28	−0.68876	0.442684	0.131829	0.502199
HbA1c	Cataracts	Weighted median	28	−0.05818	0.092272	0.528341	0.943479
HbA1c	Cataracts	Inverse variance weighted	28	0.199162	0.168736	0.237874	1.22038
HbA1c	Cataracts	Simple mode	28	−0.10213	0.142954	0.481113	0.902916
HbA1c	Cataracts	Weighted mode	28	−0.10213	0.123093	0.414003	0.902916

*P* < 0.05 was considered as significant causal relationship.

**Table 4.** The Results of Sensitivity Analysis in Reverse MR Analysis

			Heterogeneity Test			Pleiotropy Test			
Outcome	Exposure	Method	Q	Q_df	Q_pval	Egger Intercept	SE	pval	MR-PRESSO_pval
HbA1c	Cataracts	MR-Egger	220.8415	26	8.52E-33	0.062239	0.028974	0.041196	<0.001
HbA1c	Cataracts	Inverse variance weighted	260.0353	27	5.88E-40	NA		NA	

pval, *P* value.

### The Enrichment Investigation

**Figure 3.**
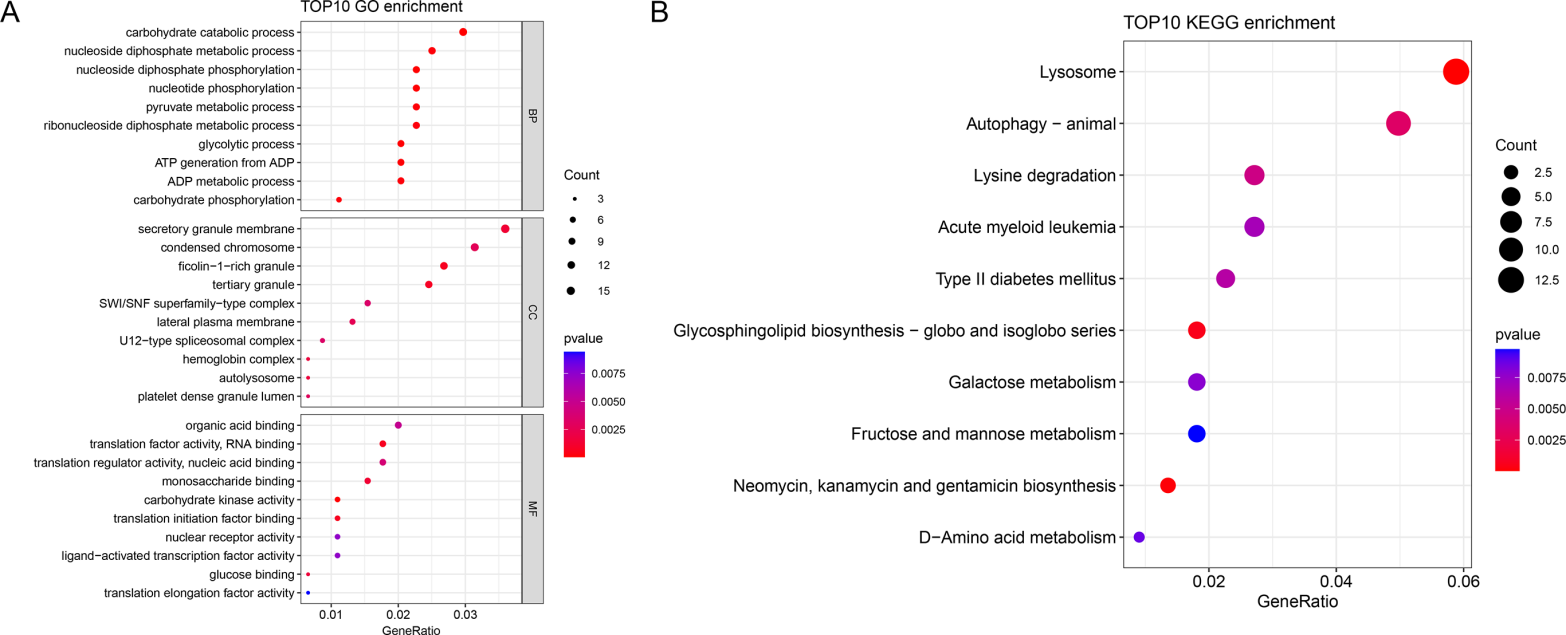
The enrichment analysis for IV-associated genes. (**A**) The TOP 10 results of GO-biological processes (BP), GO-cellular components (CC) and GO-molecular functions (MF) assembled by IV-associated genes. (**B**) The TOP 10 result of KEGG pathways enriched by IV-associated genes. The larger the node, the more genes assembled/enriched. The redder the color, the more significant the *P* value.

The enrichment analysis was performed on genes associated with IVs revealed by eQTL analysis in forward MR analyses. The results showed that these IV-associated genes were significantly enriched in GO functions associated with carbohydrate metabolism, including carbohydrate catabolic process (BP, GO:0016052) and carbohydrate kinase activity (MF, GO:0019200) (Fig. 3A). Meanwhile, these genes were mainly enriched in pathways including autophagy (KEGG: hsa04140) (Fig. 3B).

### Validation Investigation

**Figure 4.**
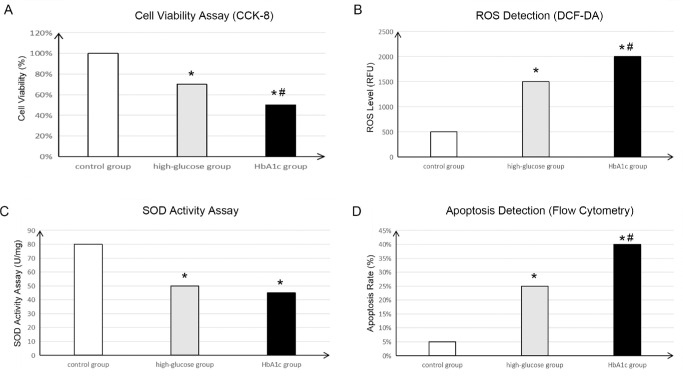
The validation analysis of HbA1c in vitro. (**A**) The cell viability investigation by using CCK-8 assay. (**B**) The ROS levels measured by DCF-DA. (**C**) The SOD activity assays to reveal the antioxidant enzyme activity. (**D**) Flow cytometry analysis to explore the cell apoptosis rate. *P < 0.05 when compared with control group; #*P* < 0.05 when compared with high-glucose group.

The validation analysis was performed based on lens epithelial cells (Fig. 4). Briefly, CCK-8 assay results indicated that cell viability in the HbA1c group was significantly lower than that in both normal control and high-glucose groups (all *P* < 0.05) (Fig. 4A), suggesting that HbA1c might inhibit lens epithelial cell proliferation and survival by exacerbating oxidative stress. Moreover, ROS levels measured by DCF-DA fluorescence probe were significantly elevated in ROS production in the HbA1c group, which was markedly higher than both the normal control and high-glucose groups (all *P* < 0.01) (Fig. 4B), indicating that HbA1c exposure elevated oxidative stress in lens epithelial cells. In addition, the SOD activity assays revealed that antioxidant enzyme activity in the HbA1c group was significantly reduced compared to the control group (*P* < 0.05). However, no significant difference was observed between two different glucose groups (*P* > 0.05) (Fig. 4C). This result suggested that HbA1c reduced cellular antioxidant capacity, thereby aggravating cell damage. Finally, flow cytometry analysis showed that the apoptosis rate in the HbA1c group was significantly higher than that in both the normal control and high-glucose groups (all *P* < 0.01) (Fig. 4D). These findings suggest that HbA1c may induce lens epithelial cell apoptosis, potentially contributing to cataract formation.

## Discussion

Although HbA1c levels are a critical component of diabetes management and has been linked to cataracts in previous studies, the exact causal relationship between HbA1c and cataract formation remains unclear.^22^ This study employed a bidirectional MR approach to investigate the causal relationship between HbA1c levels and the risk of cataracts. The forward MR analysis revealed a significant positive relationship between HbA1c levels and cataract risk, whereas the reverse MR analysis did not support a causal influence of cataracts on HbA1c levels. These findings were reinforced by sensitivity analyses and further validated by functional enrichment analysis and in vitro experiments, highlighting the importance of HbA1c as a modifiable risk factor for cataractogenesis, likely mediated by carbohydrate metabolism.

The forward MR analysis offered strong evidence that higher HbA1c levels were causally linked to a greater risk of cataract development, supporting prior epidemiological studies that had suggested a strong link between hyperglycemia and cataract formation. For instance, prior cohort studies have shown that inadequate glycemic control, reflected by elevated HbA1c levels, is linked to a notably higher risk of cataracts in individuals with type 2 diabetes.^23^^,^^24^ Similar findings were reported in a meta-analysis, which established that patients with diabetes and poor glycemic control had a higher risk of cataract development compared to those with well-controlled diabetes.^25^ It is worth noting that we used the MR-Egger method to assess the pleiotropy and heterogeneity of our model, as well as the MR-PRESSO method to calculate pleiotropy. The results indicated that the heterogeneity test showed *P* < 0.05, confirming the presence of heterogeneity. However, despite this heterogeneity, the *P* value obtained using the IVW method was less than 0.05, suggesting that the heterogeneity present in the HbA1c and cataract datasets did not significantly affect the validity of our findings. The robustness of our results could be attributed to the strength of the instrumental variables used and the consistency across multiple MR methods.^26^ These results align with our MR findings, reinforcing the conclusion that HbA1c plays a direct role in cataract formation. Mechanistically, elevated HbA1c may accelerate cataract formation through multiple pathways. A crucial pathway involves the non-enzymatic glycation of lens proteins, leading to the buildup of advanced glycation end products (AGEs) within the lens.^27^ AGEs play a role in protein aggregation and lens opacity, which are characteristic features of cataract development. Additionally, hyperglycemia increases oxidative stress within the lens, leading to cellular damage and apoptosis of lens epithelial cells.^28^ In the current study, the enrichment analysis showed that IV-associated genes were significantly enriched in carbohydrate metabolism, including carbohydrate catabolic process and carbohydrate kinase activity. Meanwhile, pathways like autophagy, which is considered a player in cell death-related process, were significantly enriched by these IV-associated genes. Our validation experiments demonstrated that elevated HbA1c levels ‌increased‌ ROS production, ‌reduced‌ SOD activity, and ‌induced‌ apoptosis in lens epithelial cells, directly supporting the hypothesis linking hyperglycemia to cataract formation. These findings align with the report of Zhai et al.^29^ that antioxidant impairment accelerates cataract progression in diabetes. Collectively, these data highlight the importance of HbA1c as a modifiable risk factor for cataracts and suggest that stringent glycemic control could play a pivotal role in cataract prevention.

In contrast, the reverse MR analysis found no evidence of a causal effect of cataracts on HbA1c levels. Although observational studies hypothesized that cataracts, particularly in advanced stages, may lead to reduced physical activity and poor access to healthcare,^30^^,^^31^ all of which could impair glycemic control, our findings suggested that cataracts themselves were not a causal factor for worsening HbA1c levels. This is consistent with a previous study that found no significant association between cataract surgery and HbA1c changes,^32^ indicating that cataract-induced visual. Our MR analysis addressed the limitations of observational studies by using genetic instruments to eliminate confounding factors and bidirectional causality, thus providing stronger evidence against a direct effect of cataracts on HbA1c levels. This insight is clinically relevant because it suggests that interventions aimed at improving glycemic control should primarily focus on metabolic factors rather than attributing worsening HbA1c levels to cataract-related limitations. Thus the absence of a causal link in the reverse direction suggested that cataracts do not directly worsen glycemic control, highlighting the need for independent management strategies for cataract prevention and glycemic control.

The study has revealed a correlation between HbA1c levels and the risk of cataracts, with potential medical applications that could be extended into the broader realm of metabolic disease management. As a stable biomarker of chronic hyperglycemia, HbA1c may serve as a cross-disease risk prediction tool for glucose metabolism-related complications (e.g., diabetic retinopathy and keratopathy). By constructing a multidimensional early-warning model combined with oxidative stress biomarkers (such as MDA and SOD), this approach could assist clinicians in identifying high-risk populations and facilitating early interventions. Secondly, the dynamic trajectory of HbA1c may reflect systemic damage caused by glucose fluctuations, a mechanism suggesting its potential as a reference indicator for personalized medication selection. For instance, patients exhibiting significant HbA1c variability could be prioritized for glucose-lowering agents with antioxidant properties. Furthermore, HbA1c quantification aids preoperative risk stratification, particularly in elderly patients or those with severe retinopathy, enabling dynamic adjustment of surgical timing and intraocular lens selection strategies based on HbA1c levels. At the public health level, the study findings support the integration of HbA1c monitoring into the preventive system for occupational eye diseases, particularly for stratified management of metabolic status among populations in specific occupations with long-term exposure to oxidative damage risks (such as ultraviolet radiation and ionizing radiation), thereby enabling multifactor collaborative prevention and control.

However, there were still several limitations that should be acknowledged in the current study. First, the research relied on genetic data from European populations, which could limit the generalizability of the results to other ethnic groups. Second, although MR minimizes confounding factors, potential bias because of weak instrumental variables or undetected pleiotropy could not be entirely excluded. Last, in the absence of direct RCT evidence, the findings of this study warranted further clinical investigation and prospective validation to ensure their applicability in clinical practice.

In conclusion, this study provided evidence supporting the causal role of elevated HbA1c levels in cataract formation, underscoring the clinical importance of tight glycemic control as a strategy for reducing cataract risk, particularly in patients with diabetes.
